# Editorial: Global excellence in natural products for endocrine disorders

**DOI:** 10.3389/fendo.2023.1325355

**Published:** 2023-11-28

**Authors:** Helena Malvezzi, Vineet K. Maurya, Eileen Brantley, Lokesh Kumar

**Affiliations:** ^1^ Hospital Israelita Albert Einstein, São Paulo, Brazil; ^2^ Department of Molecular and Cellular Biology, Baylor College of Medicine, Houston, TX, United States; ^3^ Division of Pharmacology, School of Medicine, Loma Linda University, Loma Linda, CA, United States; ^4^ GenusPlc, ABS Global, Windsor, WI, United States

**Keywords:** diabetes, glucocorticoid receptors, hormonal imbalances, metformin, hemoglobin A1c

Endocrine disorders encompass a broad spectrum of health challenges, including hormonal imbalances, metabolic syndromes and infertility in humans. It has become a significant health concern globally in recent decades, and limited therapeutic options have made it even more challenging. Therefore, it’s crucial to conduct further research to find more effective and safe therapeutic approaches to treat endocrine disorders. In this editorial, we have summarized the published articles in this special edition that explored the promising role of natural products in addressing these endocrine disorders. These natural products offer a ray of hope amidst increasing health complexities driven by modern lifestyles, dietary habits, and environmental toxins. The aim of this commentary is to highlight possible approaches to investigate numerous detrimental effects on body physiology due to endocrine disorders and to explore vulnerable targets to develop effective treatment strategies for these conditions.

Natural compounds and its structural analogues have a rich history dating back centuries, making it as a major contributor to pharmacotherapy, especially for endocrine disorders. In recent decades natural compounds are gaining renewed attention for their therapeutic potential, due to its remarkable efficacy in targeting various biological pathways, offering treatment options with minimal to no side effects, making them potential tools for managing a range of endocrine disorders, including cardiac diseases, diabetes, obesity, and infertility (**Figure 1**). Natural products encompass a diverse array of substances derived from plants, animals, and microorganisms including polyphenols, omega-3 fatty acids, probiotics, amino acids, essential vitamins, and minerals. Overall, natural products will continue to play a pivotal role in advancing drug development and addressing global health challenges, as well as achieving sustainable development goals on health.

**Figure 1:**
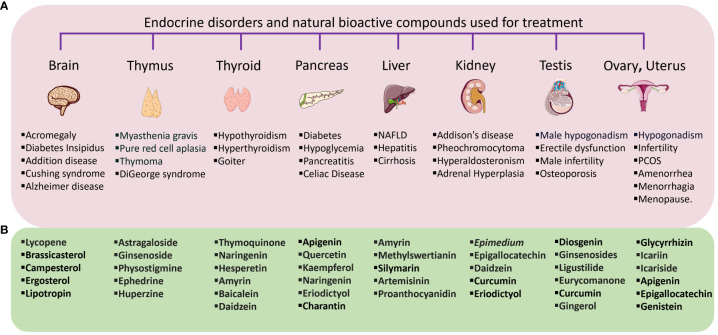
**(A)** Endocrine organs and its disorders, **(B)** Natural bioactive compounds isolated from plant extract and used for the treatment of numerous endocrine disorders of following organ.

Environmental steroids have emerged as potential endocrine disruptors, posing implications for both human health and aquatic ecosystems. The interaction between environmental steroids and the human glucocorticoid receptor (hGR) has raised concerns regarding their impact on immune and metabolic processes regulated by this receptor. However, the precise mechanisms governing their interaction with the glucocorticoid receptor in aquatic species, such as the zebrafish, remain elusive. In a featured article, Toso et al., delves into the investigation of the divergent activation of the glucocorticoid receptor in humans (hGR) and zebrafish (zfGR) by natural and synthetic steroids. Their research elucidates that while numerous glucocorticoids exhibit similar potencies as agonists for both receptors, certain synthetic glucocorticoids and mineralocorticoids manifest distinctive patterns. This observation suggests that extrapolating toxicological data from mammalian models may not consistently predict hazards in other species, such as zebrafish.

These findings emphasize the vital significance of investigating inter-species differences to enhance a comprehensive assessment of endocrine disruptor risks.

The findings of Ren et al., address the urgent global concern of obesity and fatty liver disease, conditions that have surged in the wake of high-fructose and high-fat diets. The study brings to the forefront the potential of oxymatrine, a natural compound extracted from Sophora flavescens, as a potential therapeutic agent. Oxymatrine not only alleviates obesity induced by high-fat diets but also influences the enhancer landscape of subcutaneous adipose tissues. This innovative approach sheds light on the importance of Smad3 in enhancers linked to obesity, offering a promising avenue for addressing metabolic disorders.

Siriyotha et al., investigates the benefits of multiple antihyperglycemic drugs as adjunct therapies to metformin for the treatment of cardiovascular in humans. The study directly compares the major adverse cardiovascular events associated with sodium-glucose co-transporter 2 inhibitors (SGLT2i), dipeptidyl peptidase-4 inhibitors (DPP4i), thiazolidinediones (TZD), and sulfonylureas (SUs). This study findings suggested significant reduction in cardiovascular events with SGLT2i and TZD compared to SUs as adjunct therapy with metformin. This real-world data underlines the potential benefits of these drugs in improving cardiovascular outcomes for individuals with type 2 diabetes.

In the pursuit of improved glycemic control in individuals with type 2 diabetes, Li et al., study challenges the conventional reliance on glycated hemoglobin A1c (HbA1C) as the primary determinant for initiating insulin therapy. Their comprehensive survey of real-world patients reveals that the initiation of insulin based on fasting blood glucose (FBG) or postprandial blood glucose (PBG) is a common clinical practice, particularly notable in China. This approach offers a more direct and effective method to combat clinical inertia and personalize insulin therapy according to individual needs, potentially heralding a paradigm shift in diabetes management strategies.

Chai et al.‘s meta-analysis explores the exciting area of glucagon regulation in patients with type 2 diabetes mellitus (T2DM) who are treated with DPP4 inhibitors. The results underscore the efficacy of DPP4 inhibitors in reducing postprandial glucagon levels, thereby contributing to enhanced glycemic control. This research uncovers a novel mechanism through which these drugs positively influence the management of T2DM and provides valuable insights into their potential therapeutic applications.

The broad spectrum of articles featured in this special edition highlights the crucial significance of natural products in the management of endocrine disorders. From delving into the world of environmental steroids to investigating novel compounds such as oxymatrine and rethinking strategies for insulin initiation, these studies provide innovative solutions for tackling the complex problems surrounding endocrine disorders. As we further unveil the therapeutic potential of natural products, we stand on the threshold of a new era in personalized medicine and enhanced healthcare outcomes.

## Author contributions

HM: Writing – original draft, Writing – review & editing. VM: Conceptualization, Formal Analysis, Supervision, Visualization, Writing – review & editing. EB: Writing – review & editing. LK: Supervision, Writing – original draft, Writing – review & editing.

